# Understanding the Impact of Expertise in Joint and Solo-Improvisation

**DOI:** 10.3389/fpsyg.2017.01078

**Published:** 2017-06-30

**Authors:** Johann Issartel, Mathieu Gueugnon, Ludovic Marin

**Affiliations:** ^1^Multisensory Motor Learning Laboratory, School of Health and Human Performance, Dublin City UniversityDublin, Ireland; ^2^EuroMov – University of MontpellierMontpellier, France

**Keywords:** expertise, dance improvisation, joint-action, wavelet transform, interpersonal coordination

## Abstract

Joint-improvisation is not only an open-ended creative action that two or more people perform together in the context of an artistic performance (e.g., theatre, music or dance). Joint-improvisation also takes place in daily life activities when humans take part in collective performance such as toddlers at play or adults engaged in a conversation. In the context of this article, joint-improvisation has been looked at from a social motor coordination perspective. In the literature, the nature of the social motor coordination characteristics of joint-improvisation for either the creative aspect or daily life features of this motor performance remains unclear. Additionally, both solo-improvisation and joint-improvisation need to be studied conjointly to establish the influence of the social element of improvisation in the emergence of multi-agent motor coordination. In order to better understand those two types of improvisation, we compared three level of expertise – novice, intermediate and professional in dance improvisation to identify movement characteristics for each of the groups. Pairs of the same level were asked to improvise together. Each individual was also asked to perform an improvisation on his/her own. We found that each of the three groups present specific movement organization with movement complexity increasing with the level of expertise. Experts performed shorter movement duration in conjunction with an increase range of movement. The direct comparison of individual and paired Conditions highlighted that the joint-improvisation reduced the complexity of the movement organization and those for all three levels while maintaining the differences between the groups. This direct comparison amongst those three distinct groups provides an original insight onto the nature of movement patterns in joint-improvisation situation. Overall, it reveals the role of both individual and collective properties in the emergence of social coordination.

## Introduction

Human behavior does not only consist of set goals. We plan our actions but need to constantly make changes in this plan to fit the situation requirement. At the same time, if any unplanned events emerge from our interaction with the environment, we immediately react to them. This constant interaction with the world around us is quite efficient and accurate. In other words, improvising is an action humans tend to do on a daily basis. Interestingly, we do not consider “what we do” as an improvisation. Improvisation is not a concept that is paramount to our daily thoughts even if one could consider that this is “what we actually do.” In the late 80’s, Agre and Chapman had started to question the concept of improvisation and its role in daily life activities. From their perspective, everyday is a constant moment-to-moment improvisation – “life is a continual improvisation” (Agre and Chapman, 1987, pp. 287). Usually, the term improvisation is used in arts as an off-the-cuff performance with the absence of anticipation or planning taking into account the audience (i.e., the environment). Although it is possible to improvise alone, in our everyday life, improvisation is almost all the time an action that requires an interaction either between a human and the non-human environment (e.g., synchronizing with music, with a video game and so on) or between people (e.g., collaboration, competition, and synchronization). The latter, called social interaction, is one of the most important source of improvisation. In other words, we all improvise in our daily life. In that sense, joint-improvisation can be seen as a sense of cooperation between performers (Seham, 2001) to create a moment, frequently reported as “being in the zone.” Those moments of togetherness (Hart et al., 2014; Noy et al., 2015) are the expression of integration of the individual and collective properties merged together.

The notions of individual and collective properties come from von Holst’s (1937) paper when he claimed that individual components possess intrinsic properties that tend to persist even when these components are coordinated with others (i.e., collective properties). For any biological component, there is a joint effect of the individual properties to resist to changes – maintenance tendency – in conjunction with the magnet effect attracting those components together (i.e., the collective properties). In the context of an improvisation (when movements are not constrained), one would see the individual properties as the characteristics of the performers’ creative movements whereas collective properties would be related to the interaction between these movements. In a previous study, we investigated the organization of the individual and collective properties during improvisation (Issartel et al., 2007). Participants were asked to move freely their forearm in the sagittal plane by exploring, without constraint, the whole range of frequency. Using a wavelet analysis, we found a presence of an individual motor signature expressing the intrinsic dynamic that leads the motor behavior in a specific and limited range of frequencies. However, when two people interacted together in an improvisation task, the individual motor signatures changed and were partially modulated to fit each other. More precisely, this emergence of collective properties between participants was observed in terms of frequencies of movements that could lead to coordination.

Furthermore, using the well-known mirror game paradigm (Noy et al., 2011; Gueugnon et al., 2016), Hart et al. (2014) investigated the specific moment of togetherness in improvisation. Participants were asked to mirror each other and create interesting synchronized motion with and without a designated leader. They here observed that each leader person performed a specific velocity profile of their movements (i.e., skewness and kurtosis). Interestingly, in specific moments of togetherness, both players of the interaction changed their motor signatures toward an universal signature (resembling to a velocity profile of a sine wave) in order to be coordinated and improvised together. Finally, the organization of the individual and collective properties has been extended by a recent work from Słowiński et al. (2016). They confirmed the presence of individual properties in terms of the velocity distribution of the improvised movements during mirror game. By comparing motor signatures and coordination of interactants, they showed that individual properties have to be taken into account in social coordination. Indeed, their results suggest that the similarity between individual signatures promotes interpersonal coordination during joint improvised action leading to better “social glue,” affiliation or social exchange (Wilson and Knoblich, 2005; Ashton-James et al., 2007; Semin, 2007; Hove and Risen, 2009; Miles et al., 2009; Semin and Cacioppo, 2015).

Overall, those joint-action characteristics are highly dependant upon the individual capabilities. One common way to identify the individual characteristics is to compare novice with experts. The idea is to quantify and qualify what makes an expert, the one able to perform unique, optimized, efficient, and proficient movement patterns (Kiefer et al., 2011, 2013). To characterize individual movement expertise, researchers have targeted a specific population: expert dancers. For example, Kiefer et al. (2011, 2013) have highlighted that the balance skills of expert dancer lead to greater balance ability without compromising the adaptability and flexibility of the coordinative structure. Jarvis et al. (2014) reported higher trunk variability for experts prior to landing in a “sauté” while observing a lower variability for this same group for any other kinematic and inter-segmental coordination. The above-mention results reveal the importance of the key role of individual variability when it comes down to understand movement pattern expertise. These individual characteristics were also considered in joint-action dance situations.

In joint-action situations three main characteristics could be examined: (i) subjective, (ii) physiological, and (iii) kinematic markers of joint-action. The subjective measures would tend to evaluate the sense of togetherness experienced by the performers (Nachmanovitch, 1990; Seham, 2001). Those instants, referred as “being in the zone” (in the context of an improvisation), are considered as the peak moments in terms of performance and/or synchrony amongst performers. They tend to be accompanied by physiological responses with increased heart rate associated with subjective rating of togetherness (Noy et al., 2015). The kinematic markers in joint-action also revealed that high level of togetherness between performers is characterized by smooth and symmetric movement properties (Hart et al., 2014). For example, those kinematics properties could be expressed in terms of amplitude of movement, frequencies of the movement performed or relative phase between the performers (Gueugnon et al., 2016). Along the same line, Washburn et al. (2014) have demonstrated that trained dancers have developed better visuo-motor coordination capabilities than untrained dancers. Experts express better capabilities in discriminating their partners ongoing movement and anticipating future behavior (Calvo-Merino et al., 2010). Overall, in the context of complex actor-environment interaction, experts’ better synchronization capabilities seem to play a role in activity of daily living. These capabilities would act as facilitator of social awareness and social entrainment as well as adaptive behavior.

The article investigated the question of expertise in improvisation task in the aim to specifically identify movement characteristics that would reflect expertise in dance improvisation. This identification can be done both at individual and collective levels where we expect to observe a modification of the marker of improvisation with expertise. We would then be able to question how expertise modifies the joint effect of maintenance tendency and magnet effect. The experimental manipulation of two dimensions (both individual and collective characteristics as well as expertise) will allow a double comparison of influence of an improvisation task on each of these dimensions. It will also allow us to untangle together the influence of expertise on individual and collective characteristics in improvisation task. One would expect to observe a clear difference between the levels of expertise where individual expert dancers’ movement characteristics would perform a wider variety of movements. These differences would be magnified in the context of a joint-improvisation where the magnet effect would tend to reduce the variety of movement produced for all levels of expertise while maintaining a clear difference between groups.

## Materials and Methods

### Participants

Thirty-six participants were randomly paired in 1 of the 3 specific groups of dance expertise. In the 1st group, called “Novice Dancers,” participants had no experience of dance other than what most people would have had in their personal leisure time. The second group, called “Intermediate Dancers,” had 4–5 years experience in contemporary dance. Typically, they would have attended 2–3 times of week classes while also taking part in public performances as part of a troupe. The third group called, “Expert Dancers,” had at least 10 years experience as professional contemporary dancers. Informed written consent was obtained for all participants on the day of data collection. All participants were free to withdraw from the study at any stage. Full ethical approval was granted by the University Research Ethics Committee.

### Procedure and Design

Participants were seated on a chair with their right elbow resting on a table in front of them. Participants were instructed to look at a black dot placed at eye level on a wall located 2 meters away in front of them. For all experimental Conditions, participants were asked to move their right forearm in the sagittal plane while keeping their wrist and fingers constantly aligned with their forearm (i.e., no movement of the wrist or fingers). Their left hand was resting on their left leg. Participants were instructed not to move their head or trunk and not to raise their elbow off the table. Participants were invited to freely move their forearm in the sagittal plane by exploring, without constraint, the full range of amplitude, phase, and frequency. Those free movements were performed in two Conditions (“Paired” and “Alone”). In the “Paired” Condition, participants were seated across from each other in a way that their forearms were directly aligned with the back dot located directly in front of them. In this Condition, participants were asked to take into account the movement of the other participants to perform his/her own movements. This setup was conceived to ensure that participants would only have a peripheral vision of the other participant’s forearm. In the 2nd experimental Condition, the participant was on its own, called “Alone,” where they were told, as mentioned above, to freely move their right arm in the sagittal plane. Each participant performed 1 block of 6 trials for each Condition (i.e., “Paired” and “Alone”). The Conditions were randomized across the pairs. The duration of each trial was 3 min with a 2 min rest interval between trials. The experimental set was similar to the one used in a previous article of Issartel et al. (2007).

### Materials

Elbow goniometers Biometrics SG 110 (Biometrics, Oxford, England) measured the flexion and extension of the forearm. From the elbow center of rotation, one end of the goniometer was attached to the forearm and the other end on the upper arm. The sampling rate was set at 50 Hz.

### Data Analysis

**FIGURE 1:**
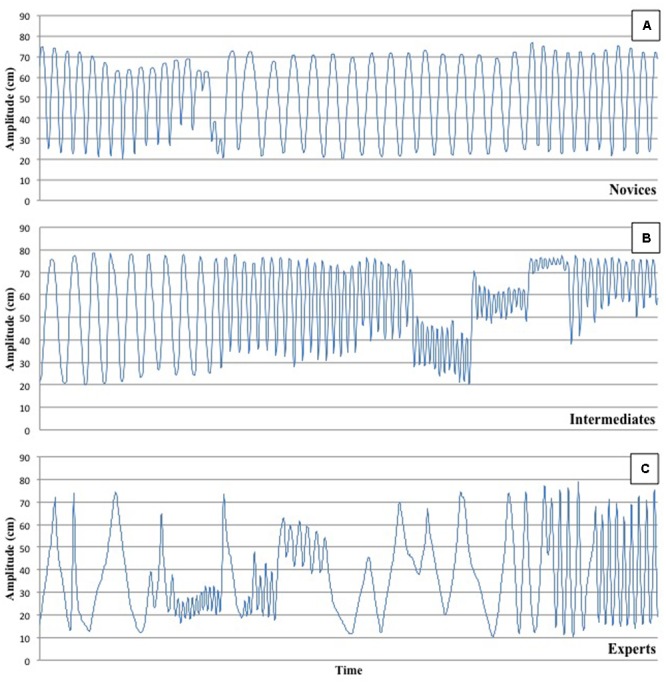
Representative example of typical movements performed by each of the 3 groups – **A** = Novices, **B** = Intermediates, and **C** = Experts. One can observe an increase of the number and spread of frequencies from Novices to Experts as well as more frequent turn over of their movements (c.f. Duration of atoms).

As participants were able to freely move their forearm, non-stationary time-series were collected preventing us from using traditional human movement signal processing methods (**Figure 1**). The method to be used had to take into account the pluri-frequency nature of the signal as well as the changes in phase that is usually observed in an improvisation-like task (see Issartel et al., 2007 for example of improvisation-like data). The wavelet transform (WT) and the cross-wavelet transform (CWT) methods were used to quantify the signals in terms of frequency and phase (Schmidt et al., 2014). Multiple frequencies can be observed at the same time and over time while also considering the relative phase for each of those frequencies. This method opens the door to multi-scale signals analyses over finite spatial and temporal domains.

The WT and CWT methods transform traditional time-series into scalograms: an expression of the signal in frequency as function of time. Those scalograms are obtained by the convolution of the time-series with an analyzing function (see Issartel et al., 2006, 2015 for more details). The scaling of this analyzing function determines the characteristic frequency of the signal at a given time. This analyzing function is also swept over time giving us an analysis of the whole time-series for a set frequency range as function of the time. To cover the frequency range of participants’ movement, the band of frequencies chosen for this analysis was [0.04–6.35 Hz]. The analysis of the signals was performed with the Morlet analyzing function (order of 8, see Issartel et al., 2006).

For the “Alone” Condition, one scalogram was analyzed as described above. For the “Paired” Condition, the CWT analysis provides us with two separate scalograms. The first one is a scalogram that is a representation of the common frequencies between the two participants. The second one represents the relative phase for each of those common frequencies.

To characterize the performance of the participants, five variables have been used. (i) We extracted the number of frequencies performed by the participants for each trial from the WT and CWT spectrum. Along the same line, (ii) we calculated the spread of the frequency range covered for each trial. The range of frequency will provide information in terms of movement speed so that we will be able to consider if some groups performed wider range of frequencies and also slower and/or faster movement. To consider the energy content of the signal, an atomic reconstruction analysis was performed. The idea was to scan the whole WT spectrum to extract specific pocket-like of events representing key moments during each trial. The reconstruction performs iterations of the spectrum to reveal the atoms containing local maxima within 1 s vicinity (Bardainne et al., 2006). The stopping criterion was set at 90% of the reconstruction level to avoid the inclusion of local maxima that would come up as mathematical artifacts of the WT and CWT analysis. Those artifacts are mainly caused by the trade-off between the accuracy in time and the accuracy in frequency that is inherent to such computation. Hence, the output from those analyses allow us to characterize (iii) the number of atoms which gives us a representation of the number of events occurring during the improvisation as well as (iv) an estimation of their duration. Finally, in order to assess coordination in the “Paired” Condition, we extracted (v) the distribution of the relative phase.

### Statistics

Five ANOVAs were applied to for the number of frequencies, the frequency range, the number of Atoms, the duration of the Atoms, and the distribution of the relative phase. Sphericity was assessed for each of these variables. When sphericity was not met, the Greenhouse and Geisser’s correction for the degrees of freedom was applied. Bonferroni’s correction *post hoc* analysis was used where necessary to assess the direction of significant effects.

## Results

### Number of Frequencies

**FIGURE 2:**
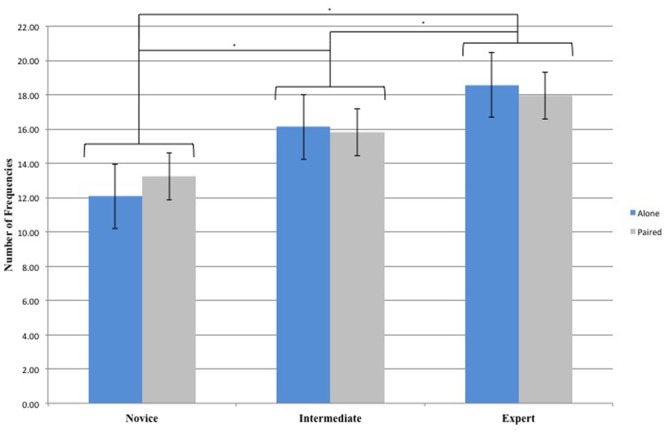
Number of Frequencies for the 3 Groups (Novice, Intermediate, and Expert) and the 2 Conditions (Alone and Paired). ^∗^Asterisks indicate significant differences *P* < 0.05.

The 3 (Groups) × 2 (Conditions) repeated-measures ANOVA on Number of Frequencies yielded a significant main effect for Groups [*F*(2,33) = 19.83, *p* < 0.01, $\eta_{p}^{2}$ = 0.55]. There was no main effect for Conditions [*F*(1,33) = 0.6, *p* > 0.05, $\eta_{p}^{2}$ = 0.02] and no interaction effect between Conditions and Groups [*F*(2,33) = 0.46, *p* > 0.05, $\eta_{p}^{2}$ = 0.03]. *Post hoc* comparisons revealed significant differences between Novice and Intermediate Dancers (*p* < 0.01), Novice and Expert Dancers (*p* < 0.01), and Intermediate and Expert Dancers (*p* < 0.05) revealing that Intermediate Dancers performed more frequencies than Novice Dancers and that Expert Dancers performed more frequencies than Intermediate and Novice Dancers (**Figure 2**).

### Spread of Frequencies

**FIGURE 3:**
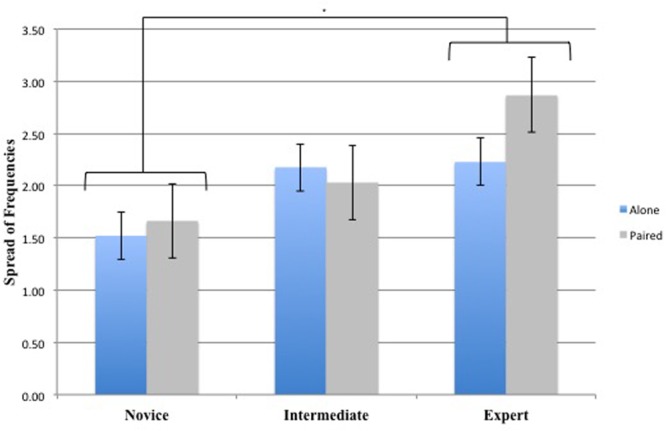
Spread of Frequencies for the 3 Groups (Novice, Intermediate, and Expert) and the 2 Conditions (Alone and Paired). ^∗^Asterisks indicate significant differences *P* < 0.05.

The 3 (Groups) × 2 (Conditions) repeated-measures ANOVA on Spread of Frequencies yielded a significant main effect for Groups [*F*(2,33) = 7.71, *p* < 0.01, $\eta_{p}^{2}$ = 0.32]. There was no main effect for Conditions [*F*(1,33) = 1.32, *p* > 0.05, $\eta_{p}^{2}$ = 0.04] and no interaction effect between Conditions and Groups [*F*(2,33) = 1.56, *p* > 0.05, $\eta_{p}^{2}$ = 0.09]. *Post hoc* comparisons revealed significant differences between Novice and Expert Dancers (*p* < 0.01) revealing that Expert Dancers explored a larger range of frequencies in comparison with Novice Dancers (**Figure 3**). There were no significant differences between Intermediate and Novice Dancers (*p* > 0.05) or Intermediate and Expert Dancers (*p* > 0.05) indicating that the Intermediate Dancers behavior is situated between the Novices and the Experts Dancers.

### Number of Atoms

**FIGURE 4:**
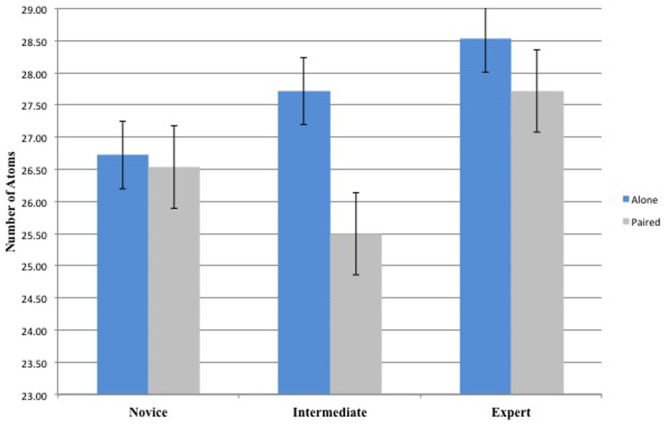
Number of Atoms for the 3 Groups (Novice, Intermediate, and Expert) and the 2 Conditions (Alone and Paired).

The 3 (Groups) × 2 (Conditions) repeated-measures ANOVA on Number of Atoms did not yielded any significant main effect for Conditions [*F*(1,33) = 1.54, *p* > 0.05, $\eta_{p}^{2}$ = 0.05] or Groups [*F*(2,33) = 1.45, *p* > 0.05, $\eta_{p}^{2}$ = 0.08]. Also, there was no interaction effect between Conditions and Groups [*F*(2,33) = 0.41, *p* > 0.05, $\eta_{p}^{2}$ = 0.02]. This result indicates that the expertise level does not influence the number of events performed by the participants (**Figure 4**).

### Duration of Atoms

**FIGURE 5:**
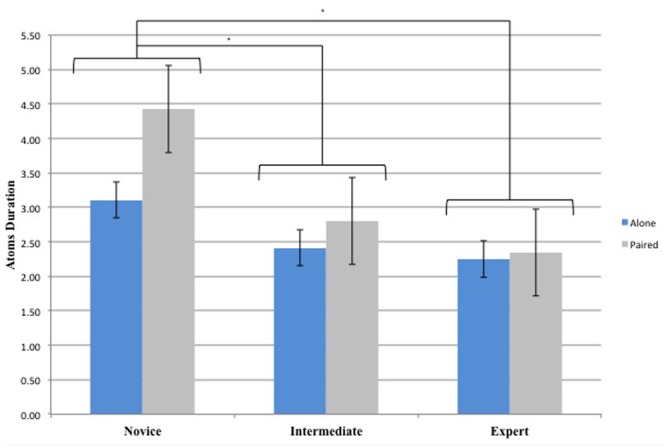
Duration of the Atoms for the 3 Groups (Novice, Intermediate, and Expert) and the 2 Conditions (Alone and Paired). ^∗^Asterisks indicate significant differences *P* < 0.05.

The 3 (Groups) × 2 (Conditions) repeated-measures ANOVA on Atoms Duration yielded a significant main effect for Groups [*F*(2,33) = 15.34, *p* < 0.01, $\eta_{p}^{2}$ = 0.48]. There was main effect for Conditions [*F*(1,33) = 9.94, *p* < 0.01, $\eta_{p}^{2}$ = 0.23] and an interaction effect between Conditions and Groups [*F*(2,33) = 3.7 *p* < 0.05, $\eta_{p}^{2}$ = 0.18]. *Post hoc* comparisons indicated significant differences between Novice and Intermediate Dancers (*p* < 0.01), Novice and Expert Dancers (*p* < 0.01) for both Conditions revealing that both Intermediate and Expert Dancers tend to perform each atom for a shorter duration in comparison with Novice Dancers (**Figure 5**). Also Novice Dancers in the Alone Condition perform each movement for a longer period of time in comparison with the Paired Condition (*p* < 0.01). At Condition level, there was no significant difference between Intermediate and Expert Dancers (*p* > 0.05).

### Distribution of the Relative Phase

**FIGURE 6:**
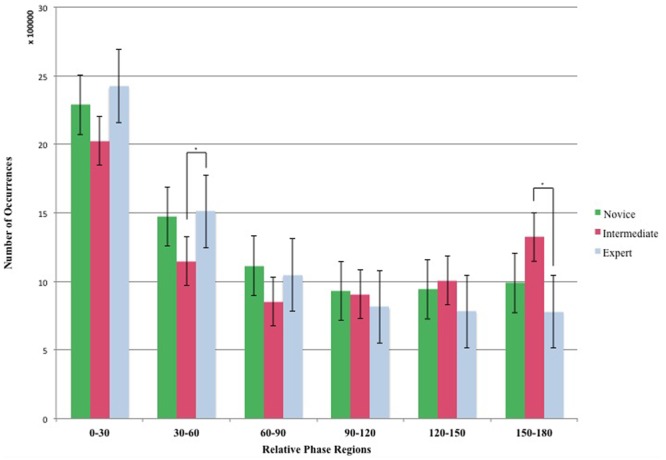
Number of Occurrences of the Relative Phase Regions for the 3 Groups (Novice, Intermediate, and Expert). The relative Phase is distributed in six regions from 0° to 180°. ^∗^Asterisks indicate significant differences *P* < 0.05.

The relative phase values were extracted from the CTW spectrum. The distribution of the relative phase angles was determined across six 30° regions of relative phase between 0° and 180°. A 3 (Groups) × 6 (Phase regions) ANOVA yielded a significant group difference for the 30°–60° region [*F*(2,15) = 4.61, *p* < 0.05, $\eta_{p}^{2}$ = 0.49] and for the 150°–180° region [*F*(2,15) = 14.87, *p* < 0.05, $\eta_{p}^{2}$ine-formula> = 0.58]. *Post hoc* analyses revealed two significant differences between Intermediates and Expert Dancers. Firstly, Expert Dancers explored the 30°–60° region more often than the Intermediate Dancers. Secondly, results suggest a higher entrainment of Intermediate Dancers toward the anti-phase region (150°–180° region) in comparison with the Expert Dancers. No other significant differences were found (**Figure 6**).

## Discussion

This study had the objective to investigate the movement characteristics reflecting the expertise in dance improvisation. Three level of expertise were considered (novice, intermediate, and expert dancers). To identify the individual characteristics, each of the dancers performed an improvisation on their own. To analyze the collective properties, dancers performed an improvisation task in pairs. The results clearly show a pathway from novice to experts when it comes down to define the type of movement performed by dancers. This pathway was found in both individual and collective improvisation.

When scrutinizing the experts specific behavior, the larger number of frequencies (**Figure 2**) performed illustrate a richer movement production as they explore a larger and more spread range of frequencies. In other words, they can produce, a wider range of actions while also exploring more frequencies within this wider spectrum. Experts perform slower movement (lower frequencies) in comparison with novices and intermediates. It is important to highlight that in term of “difficulty/complexity” those movements could have been performed by novices and intermediates. There is no mechanical, physiological or neuromuscular constraints that could explain the absence of certain type of movement. This observation crystallized the unique capability of expert dancer to produce, on their own, but also in the interaction with others, certain movements that everyone could perform but that only experts actually perform. In other words, everyone is capable of performing this wide range of action but only expert manage to explore it in the context of this improvisation. This trait is central in our understanding of dance expertise, and more widely in our understanding of movement expertise in general. Expert dancers are able to produce a unique motor performance within the same range of possibilities available to novice and intermediate dancers. Experts and Intermediates dancers also tend to move on, from one type of action to the next one, more often than Novice dancers (i.e., shorter atom duration) while going though intermediate phases that lead to the next phase of joint-action. Overall, those findings demonstrate that the amount of experience in moment-to-moment improvisation enhances the capability and capacity of the performers.

As classicially reported in the literature, behavioral synchrony has been described as a marker of expertise (Noy et al., 2011; Sofianidis et al., 2012, 2014; Washburn et al., 2014). Expertise can be qualify as an ability to be more tuned with the “information about sequence structure and upcoming movement possibilities” (Washburn et al., 2014, p. 11). Better ability to distinguish grammatical sequence (Opacic et al., 2009), better at reading current and future events. It’s an ability to jointly consider the performer own movement capabilities and the expectation of the confederate own capabilities. Dance expertise favors the emergence of moment-to-moment coupling (in both frequency and phase) and better movement discrimination such as deciphering what their partners would perform while also been able to anticipate future events. This will in turn facilitate the synchronization between the performers (Calvo-Merino et al., 2010). Those two elements: anticipation and discrimination of the moment-to-moment coordinated performance would occur concomitantly in an improvisation task. The interaction between anticipation and discrimination can be discussed in line with the concepts of maintenance tendency and magnet effect. Being able to discriminate his/her partner’s movement would in turn facilitate the magnet effect and therefore the social entrainment between the two performers. At the same time, being able to better anticipate their partner’s movement would enhance the performer’s choice of action to be performed. Then maintenance tendency would be at play guiding the performer to continue to explore with his/her own individual movement characteristics (von Holst, 1937). In other words, the more the dancers anticipate, the more they can keep their own motor signature. It is the same principle when a couple of salsa dancers are perfectly in phase but the woman partner add extra little moves with her head or leg. It is because she anticipates the movement of her co-actor, that she can maintain her own motor signature and add other ancillary movements. In addition, when the woman dancer is able to anticipate, the male dancer is more incline in maintaining his own performance (maintenance tendency). This point is in a way contradicting Washburn et al. (2014)‘s argument as they suggest that dancers higher level of coordination could be either due to a better ability at (i) discriminating movement properties or (ii) at anticipating confederate actions independently of their own action capabilities. Based on the specific expert behavior observed in this study, expert improvisation seems to reflect the conjunction of the individual and collective properties (the alliance of maintenance tendency and magnet effect) rather than a dissociation between the performer’s action capability and their ability in discrimination and anticipating the action of others.

The unique characteristics of expertise can also be interpreted in terms of expert ability to optimize task’s constraint (Newell, 1986; Sofianidis et al., 2012), enabling the emergence of complex physical movement (Kiefer et al., 2011). Also as proposed by Sofianidis et al. (2015, p. 216) expert dancers may have an “improved multisensory integration capacity.” The authors made this discussion point in the context of an interpersonal ankle/hip synchronization task where expert dancers depicted a more stable ankle/hip phase relationship. The expertise unique characteristics observed in our study are in line with Sofianidis et al. (2015) findings and those of Washburn et al. (2014) described above. On one hand, expert dancers have the capacity to produce unique movements while taking into account the movements proposed by their partner. The observed coordinated behavior reflect the combination of their own movement capabilities, their ability to discriminate the information of the confederate action while also anticipating future movements. On the other hand, novices were less capable of anticipating, discriminating while also having reduced movement capabilities resulting in a reduced variety of movement, a lower range of frequencies and a tendency to maintain longer any performed frequency.

As for intermediate participants, it seems they are “on the way of becoming expert” in the sense they do not behave as novices but they are not yet experts, when observing all key variables. However, the relative phase results are unique and raise an interesting discussion point. Intermediate dancers manage to explore more the anti-phase region than both experts and novices. Why aren’t expert using this kind of coordination? Is it a lack of expertise? This argument does not appear to be very convincing as the experts have five more years of experience. They have been employed by professional choreographers for years to create and performed public performances. If their expertise is not a reason explaining those differences, then we should consider the nature of the relationship between frequency and relative phase in movement production. To contextualize this interaction, it seems important to make a reference to the HKB Model (Haken et al., 1985) demonstrating that a modification of the control parameter (e.g., frequency) alters the order parameters (e.g., relative phase). More specifically in Bardy et al.’s (2002) experiment participants, stood in front of a large video screen and were asked to track the front-to-back oscillations of a video graphic target that varied in frequency in a stepwise manner. The authors observed a qualitative change of the order parameter (the relative phase between the ankle and the hip) due to the increased frequency of target motion. In the context of this current improvisation task, we have observed that expert dancers proposed a larger range of movement frequencies as well as a higher number of frequencies. Those unique frequencies only developed by experts, seem to characterize dance expertise. As a consequence, it seems possible that this unique set of frequencies have on knock-on effect in their ability to also propose a wide range of relative phase (even non-natural ones when performing 30°–60° relative phase). This argument is in line with the performance of the intermediate dancers. This group performed more anti-phase movement than the expert dancers while been unable to perform the same range of frequencies in comparison with the expert group. This finding opens the doors to future research: could practice/learning bring the expert dancers to the next level where they would be able to maintain their range of movement frequencies while performing more anti-phase coordination? Likewise, would expert dancers be better at coordinating in an unusual range of relative phase (30°–60°) that can only be possible after learning such a non-spontaneous range of coordination (Zanone and Kelso, 1992)?

Overall the improvisation situation proposed in this study revealed that expert dancers are able to come up with a unique creative performance through movement patterns in space and time. Not only those creative performance characteristics are present in a solo improvisation; unique expertise trait were also found in the joint-improvisation. Results of this study revealed that experts developed specific non-verbal communication, through their unique movement patterns, as observed with the behavioral markers discussed above. Expert dancers are attuned to their own movement patterns (Opacic et al., 2009) and also those of their partners during a creative performance. This acquired double propensivity to perform a unique set of movement while taking into account the confederate’s movement seems to be a signature of dance experts in the context of a joint-improvisation. Overall, better social coordination ability coupled with higher action capabilities (and/or creativity) could enhance daily life social activities in increasing cohesion and communication (Dale et al., 2014). In that sense, this expertise could also bring a better adaptive behavior in the work place and/or during any type of group physical activities.

## Ethics Statement

All authors acknowledge ethical responsibility for the content of the manuscript and will accept the consequences of any ethical violation. This work received full ethical approval from University of Montpellier (France).

## Author Contributions

JI and LM conceived and designed the experiment. JI performed the data collection and data analysis. JI, LM, and MG wrote the article.

## Conflict of Interest Statement

The authors declare that the research was conducted in the absence of any commercial or financial relationships that could be construed as a potential conflict of interest.
